# A Novel Approach to Cure-on-Demand Coatings Using Ammonia to Catalyze Thiol-Acrylate and Thiol-Epoxy Reactions

**DOI:** 10.1021/acsomega.5c00523

**Published:** 2025-05-15

**Authors:** Md Abdullah Al Mahmud, John A. Pojman

**Affiliations:** Department of Chemistry and the Macromolecular Studies Group, 5779 Louisiana State University, Baton Rouge, Louisiana 70803, United States

## Abstract

Cure-on-demand thiol-acrylate and thiol-epoxy coatings were developed by using ammonia as a catalyst. This novel method is a one-pot formulation that eliminates the need for volatile organic components and introduces a novel curing technique for coatings. Two ammonia sources were employed: a 30% aqueous solution of ammonia and ammonia generated by the urea-urease clock reaction with watermelon seed powder (WMSP) serving as a source of the urease enzyme. The pot lives were extended to at least 30 days by adding stabilizers. The use of the urea-urease clock reaction produced ammonia after a programmable delay, which allowed the coating to be covered with aluminum foil to prevent ammonia loss. The cure times for thiol-acrylate coatings were shorter, although their mechanical strengths were lower compared to thiol-epoxy coatings, which had longer cure times but superior mechanical properties.

## Graphical abstract

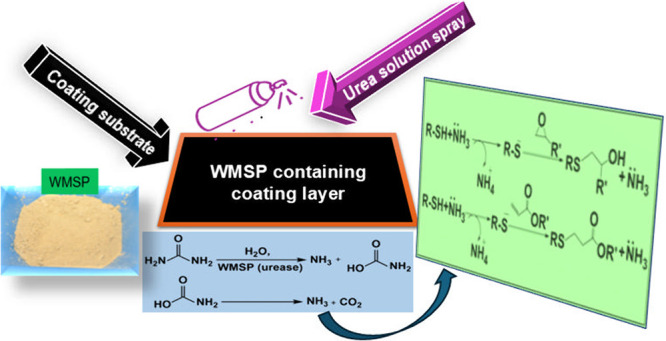

## Introduction

Thiols used in polymer chemistry have gained significant interest in the last 20 years due to their effectiveness and adaptability. Thiol chemistry can be split into two groups: radical-mediated reactions connected to the thiol-ene and thiol-yne reactions and base-catalyzed nucleophilic reactions associated with the thiol-epoxy, thiol-isocyanate, thiol-*N*-acrylamide and thiol-acrylate Michael addition reactions.^1–15^ Among these, the Michael addition reaction has been investigated for coating applications since the 1980s.^16^ Due to the benefits of thiol-click chemistry, nucleophilic thiol reactions, such as base-catalyzed thiol-epoxy, thiol-isocyanate, and thiol-acrylate Michael addition reactions, have been successfully employed to quickly and efficiently create cross-linked polymers.^17–21^ The flexibility of the weak sulfur–hydrogen bond makes it possible to start the thiol-epoxy and thiol-Michael addition reaction using a variety of catalysts.^3,22,23^ Bases catalyze thiol-Michael additions.^18,24^ For example, Khan et al. created thiol-acrylate hydrogels for studying cancer tumor growth.^25–27^ They utilized sodium hydroxide to raise the pH and deprotonate the thiol, converting it to a thiolate anion, which then reacts with the acrylate.

In this paper, we describe one-pot, solvent-free coating formulations of a trithiol with epoxy resins and a trithiol with acrylates. Both coatings from the thiol-epoxy and thiol-acrylate reactions were catalyzed by ammonia. We initially used a 30% aqueous solution of ammonia, which could lead to the loss of the catalyst by evaporation of the ammonia that could create health and environmental risks.^28^ To avoid such loss, we switched to a tunable approach that produces ammonia in situ. Our laboratory has pioneered the use of pH clock reactions to trigger polymerization and gelation. Such reactions exhibit a large pH change after a programmable “clock time”. Our first system was a formaldehyde clock reaction to trigger the thiol-acrylate Michael addition.^29^ To avoid the toxicity of formaldehyde, we switched to a urea-urease system. The hydrolysis of urea to produce ammonia and carbon dioxide is catalyzed by urease (Figure 1). Hu et al. showed that the production of base and the pH dependence of the urea-urease reaction give rise to base-catalyzed feedback.^29–31^

**Figure 1:**
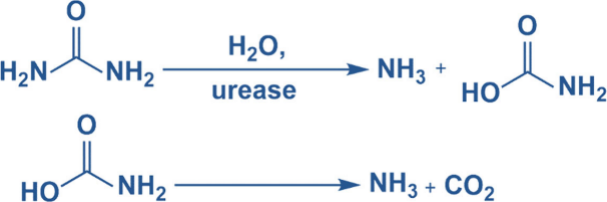
Hydrolysis of urea in the presence of urease enzyme.

Jee et al. showed that the urea-urease reaction could be used to create a time-lapse polymerization of an aqueous thiol-acrylate solution.^32^ They proposed that it was NH_4_OH that catalyzed the thiol-acrylate reaction. Pojman received a patent on this time-lapse polymerization approach.^33^ Mai et al. demonstrated that an extract from watermelon seeds provides a stable form of urease.^34^ This watermelon seed powder (WMSP) contains urease, which is stable for at least 1 year under ambient conditions. Bashir et al. used the watermelon seed powder and urea to control the gelation of poly­(vinyl alcohol) with borate.^35^

Ammonia can serve as a catalyst for the addition of a thiol to an acrylate and for the reaction of a thiol with an epoxy. A 30% solution of aqueous ammonia contains a small fraction of NH_4_OH; almost all the nitrogen is present as NH_3_.^24^ Directly applying aqueous ammonia solution to a solution of a thiol and acrylate or a thiol and epoxy will result in a curing of the layer as the ammonia diffuses into the layer. This approach is similar to vapor injection curing (VIC) with a gaseous amine to cure polyurethane coatings.^36^ However, VIC requires a closed chamber and a method to safely capture the amine after the reaction is complete.

To avoid the potential hazards of ammonia vapors from using a 30% solution of aqueous ammonia, we explored applying an acidic solution of urea onto the resin formulation containing WMSP. The conversion of urea to ammonia by urease was delayed by the low pH, so a time delay was created to allow the coating to be covered with aluminum foil before the ammonia was produced. This work was designed with a specific application goal, building upon the research of Gary et al. on cure-on-demand non-skid coatings achieved through frontal polymerization.^37^

## Materials and Methods

Trimethylolpropane triacrylate (TMPTA), Ebecryl 605 and pentaerythritol triacrylate (PETIA) were purchased from Allnex (Alpharetta, GA). Trimethylolpropane tris­(3-mercaptopropionate) (TT1), phenylphosphonic acid (PPA), and bisphenol A diglycidyl ether (BADGE) were purchased from TCI America, and trimethylolpropane triglycidyl ether (TMPTE) was purchased from Sigma-Aldrich. 4-Methoxyphenol (MeHQ) was obtained from Sigma-Aldrich. Urea was purchased from AC Alpha Chemicals, and hydrochloric acid was purchased from BDH Chemicals. Crimson Sweet Watermelon Seeds were purchased from EDEN BROTHERS. Fumed silica (FS) (Aerosil 200, 175–225 m^2^ /g BET surface area) was obtained from Evonik Industries (Parsippany, NJ). Zoltek PX35 (referred to as Zoltek or milled carbon fiber (CF), 150 μm length × 7.2 μm diameter) was provided by Zoltek Companies, Inc. (St. Louis, MO). Aluminum oxide (16 grit, 14 mesh, 1.2 mm) was obtained from Floorguard Products, Inc.

## Formulation and Curing Technique

The formulation was designed by using some fillers along with chemicals (Figure 2) so that the coatings could be applied to the substrate without the formulation flowing or the fillers settling. The thiol-epoxy coating formulation was fabricated using a 1:1 stoichiometric ratio of thiol groups to epoxy groups, and the same ratio was used for the preparation of thiol-acrylate coatings. All additional components were added in parts per hundred resins (phr), which is the amount of material added (in grams) for every 100 grams of resin.

**Figure 2:**
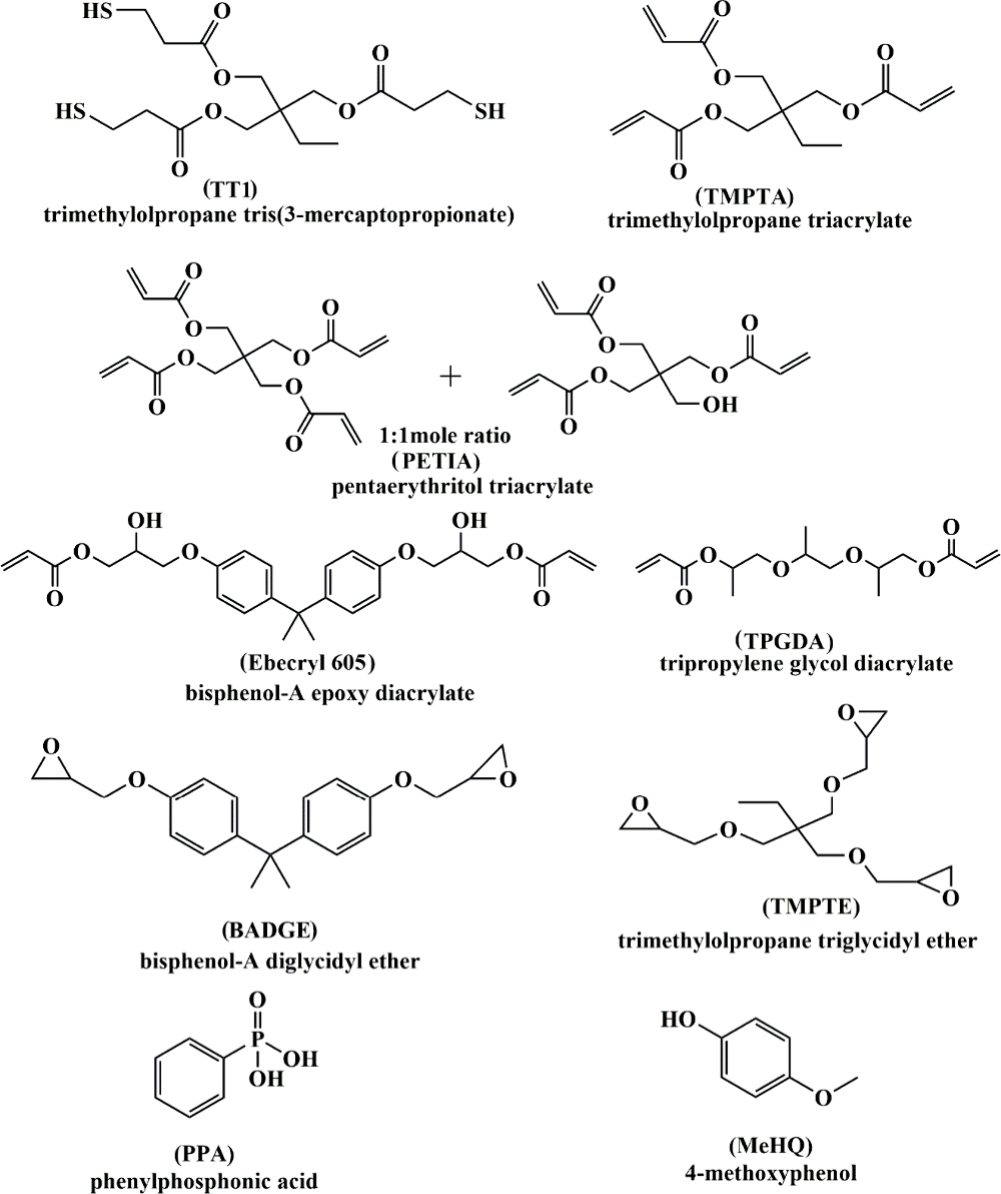
Structures of the chemicals used.

The primary issue that these formulations faced was their short pot lives. To stabilize the pot lives of these formulations, 4-methoxy phenol (MeHQ) was added to inhibit the carbon-centered radical polymerization of acrylates, and phenylphosphonic acid (PPA) was added to suppress the deprotonation of thiols.^38^ To increase the viscosity, fumed silica was added to the formulation. Aluminum oxide was added to the formulation, followed by milled carbon fiber. Milled carbon fiber and Al_2_O_3_ were added for enhancing mechanical performance, as well as abrasion, corrosion, and thermal resistance.

The thiol-acrylate and thiol-epoxy coating formulations can be cured using either an aqueous solution of ammonia or a urea-urease reaction. We developed five different formulations using three acrylates and two epoxies. For in situ ammonia production, we created other formulations. We used watermelon seed powder (WMSP) as a source of urease enzyme to produce in situ ammonia (Figure 1) for curing both the thiol-acrylate and thiol-epoxy coatings. This urea-urease reaction can be carried out in several ways: by mixing WMSP with the resins and spraying a urea solution or acidic urea solution (pH ∼3–4); by combining both WMSP and urea with the resins and spraying only water or acidic water (pH ∼3–4); by mixing urea with the resins and dispersing WMSP on top of the coatings; or by dispersing WMSP on top of the coatings and spraying a urea solution over it. However, combining WMSP with thiol-epoxy did not work. After a few hours, the WMSP was inactive. We speculate that the epoxy resin reacted with urease and destroyed its activity. Therefore, the thiol-epoxy formulation required WMSP to be dispersed on top of the coatings.

We conducted a study with the formulations listed in Table 1b. 1 phr watermelon seed powder (WMSP) was used as a source of urease enzyme, and a 20% (w/w) aqueous acidic (pH ∼4) urea solution was sprayed on top of the coatings on the substrate. If all the urea was converted to ammonia, the concentration was [NH_3_] = 4.54 M. We also conducted another study by spraying 30% aqueous solution of ammonia ([NH_3_] = 14.8 M). We also conducted several trials using varying concentrations of urea solution and WMSP to investigate their effects on cure time for both thiol-acrylate and thiol-epoxy coating formulations. The goal was to increase ammonia production and accelerate the cure time as the urea and WMSP concentrations increased. Urea concentrations ranged from 5% to 54% (w/w), while WMSP concentrations ranged from 0.5 to 8 phr.

**Table 1:** Pot Lives of Thiol-Acrylate and Thiol-Epoxy Formulations

a) Pot Life without Stabilizers	
Formulations (1:1 Stoichiometric Mixture of Resins)	Pot Life
TMPTA-TT1	<15 min
PETIA-TT1	<5 h
Ebecryl 605-TT1	<5 h
BADGE-TT1	<20 days
TMPTE-TT1	<30 days

b) Pot Life after Adding Stabilizers		
Formulation (1:1 Stoichiometric Mixture of Resins)	Stabilizers	Pot Life (Days)
TMPTA-TT1, 4 phr fumed silica, 22 phr milled carbon fiber, and 66 phr aluminium oxide	0.5 phr MeHQ and 0.3 phr PPA	500
PETIA-TT1, 4 phr fumed silica, 20 phr milled carbon fiber, and 66 phr aluminium oxide	0.5 phr MeHQ and 0.3 phr PPA	430
Ebecryl 605-TT1, 3 phr fumed silica, 18 phr milled carbon fiber, and 66 phr aluminium oxide	0.6 phr MeHQ and 0.4 phr PPA	270
BADGE-TT1, 3 phr fumed silica, 18 phr milled carbon fiber, and 66 phr aluminium oxide	0.4 phr PPA	<30
TMPTE-TT1, 4 phr fumed silica, 20 phr milled carbon fiber, and 66 phr aluminium oxide	0.4 phr PPA	<60

The loss of ammonia was prevented by covering the coatings with aluminum foil (Figure 3c). As the ammonia gas diffuses into the layer, it is supposed to deprotonate the thiol, allowing the addition to the acrylate or epoxy resins. The results from other ammonia producing techniques using WMSP and urea will also be discussed in the Results and Discussion section.

**Figure 3:**
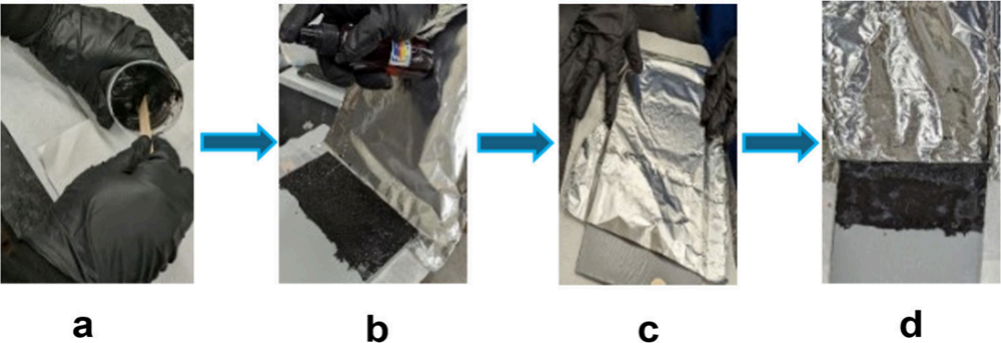
Application and curing strategy of the formulation. (a) A small jar contains coatings and a wooden tongue to mix the formulation manually. (b) Coatings applied on the metal substrate and spraying urea solution on top of it. (c) Covered with aluminum foil to prevent loss of NH_3_. (d) Sighting of the foil after curing the coatings.

Tests were typically performed at room temperature. However, some samples were examined at −5, 5, and 50 °C. The thickness of the coatings was approximately 1 mm. To assess the status of the coating’s curing process manually, pressure was applied to the surface using a finger on top of aluminum foil. If the surface could not be depressed, then it was assumed that the coating had cured. The curing was confirmed by the disappearance of the monomer’s characteristic functional groups using IR spectral analysis.

## Adhesion Performance on Substrates

Adhesion of coatings to the substrate is of prime importance. As a result, several substrates were tested for proper adhesion of all of the coating formulations. We tested steel panels coated with an epoxy primer, bare cold-rolled steel panels, brick, concrete, copper sheet, balsa wood, PVC plastics, and untreated aluminum. All of the experiments were carried out under ambient conditions. In this study, we have included all the characterization results from using different sized steel panels coated with an epoxy primer (Interbond 998, from International Paint).

## Analysis of Monomer Conversion Percentage by the FTIR Technique

The conversions of thiol, epoxy, and acrylate groups were confirmed by using a Bruker Tensor 27 Fourier transform infrared (FTIR) spectrometer fitted with a Pike Miracle single-bounce diamond attenuated total reflectance (ATR) cell. The data was collected between 400 and 4000 cm^–1^, with a resolution of 4 cm^–1^. 32 scans were done for each spectrum, and all samples were solid polymer. Opus, Bruker’s FTIR software program, was used to process the FTIR data. The thiol group concentration was tracked using the S—H stretching peak, which has a center point at around 2571 cm^–1^, and the acrylate group concentration was found using the C=C stretching band, which has a center point at around 1630 cm^–1^. Epoxy group’s C—O stretching band was detected at around 910 cm^–1^.^39,40^ We considered the carbonyl group’s (C=O) peak at 1720 cm^–1^ as a reference.

$$\mathrm{Percentage Conversion}=\left(1-\frac{\frac{M_{a}}{M_{ac}}}{\frac{M_{b}}{M_{bc}}}\right)\times 100\text{\%}$$

*M*_*b*_ is the monomer peak area before curing, and *M*_*a*_ is the peak area after curing. *M*_*bc*_ is the carbonyl group’s peak area before curing, and *M*_*ac*_ is after curing. To calculate the percentage of functional group conversion, the resin’s FTIR peak areas were compared with the areas of cured polymer peaks.

## Mechanical Performance Tests on Cured Coatings

### Pencil Hardness Test

The pencil hardness tests were performed according to ASTM D6336-22. A pencil hardness tester (GLTL, Germany-STAEDTLER) with a load of 500 g was used for this test, and wood pencils with hardness levels ranging from HB (the softest pencil) to 6H (the hardest pencil) were used. The pencil lead was rubbed at a 90° angle with abrasive paper until a flat, smooth, and circular cross section was produced. The pencil hardness tester’s fixed slot held the sharpened pencil against the coating at a 45° angle. The pencil hardness tester was placed on the coated surface and pushed away from the operator with a stroke of 6.5 mm. The surface was then examined for scratches and gouges. The test was conducted for each sample by starting with the hardest pencil and continuing down the hardness scale until two end points were reached. The coating’s “pencil scratch hardness” refers to the hardness of the pencil that could not leave a visible scratch on the coating, while the coating’s “pencil gouge hardness” refers to the hardness of the pencil that could not leave a cut or gouge in the coating. This test was repeated three times.

### Shore Hardness Test

To examine mechanical performance in depth, we performed shore hardness tests in accordance with ASTM D2240, using a durometer. There are two forms of shore hardness: shore A and shore D. Shore D refers to rigid polymeric materials with scales ranging from 0 to 100D. A hand-held digital durometer, model number 560-10D, manufactured by Gain Express Holdings Ltd., was utilized. According to ASTM D2240, the coating thickness must be at least 6 mm, and the tests were performed five times on each sample while preserving a 6 mm distance from the test spot. By stacking 2-mm samples three times, the samples were thickened to a 6 mm thickness.

### Impact Test

Impact testing was performed in accordance with the ASTM G14 guidelines. A 1.81 kg portion was dropped onto an indenter. Each impact test involved 25 impacts in a 7 × 7 cm^2^ area. Next, any coating that had become loose around the impact zone was removed with a 1-inch chisel. The number of links among adjacent effects was counted. The score was calculated by multiplying the sum of the connections by 2.5 and subtracting it from 100. The percentage of undamaged coating that is still present between the impact locations is reflected in the overall score.

### Cross Hatch Adhesion Test by Tape

The cross hatch adhesion test was performed according to the ASTM D3359 guidelines. This adhesion test was conducted to assess the adhesion of the thiol-epoxy and thiol-acrylate coatings to the substrates used in this study. Adhesion was evaluated qualitatively on a scale of 0 to 5 after an X-cut was made through the film to the substrate; pressure-sensitive tape was put over the cut, and it was then removed.

### Chemical Resistance Test

The relative resistance of coatings against a variety of chemicals, such as ethanol, natural saltwater, motor oil, detergent, and deicing/defrosting fluid, was ascertained through qualitative chemical testing. Each coating was placed in a 1,400 mL beaker and halfway submerged in ethanol for 24 h, deicing/defrosting fluid for 24 h, and then seawater, motor oil, or detergent for 4 weeks. The beakers were then sealed with aluminum foil. Following removal, a 2.5 cm chisel was used to check for coating softening or loss of adhesion on each coating substrate. Together with the impacted versus non-impacted coating, the unsubmerged and immersed portions were compared. Prior to examination, the coatings immersed in ethanol and deicing/defrosting solution were allowed to recover for 6 h.

### Thermal Stability Test

A thermal stability test was performed for cured thiol-acrylate and thiol-epoxy coatings under nitrogen purge at a rate of 100 mL min^–1^, 50 °C min^–1^ heating rate up to 600 °C with a thermal gravimetric analyzer (model TGA 550). TA Instruments’ TRIOS software was used to analyze the sample’s degradation behavior for all the cured samples. Each cured coating sample was subjected to a thermal stability test at 50 °C for three months in an oven to assess any potential deformation.

## Results and Discussion

### Pot Life Stabilization and Curing of Formulations

To be of practical use, our formulations must have a long pot life after mixing. We studied methods to stabilize the formations. Following the successful stabilization of the samples with the addition of essential components, samples from each formulation were cured.

Table 1a shows the pot lives of formulations without stabilizers.

Thiol-acrylate formulations have a limited shelf-life due to several reasons.^41^ These include the reaction of peroxides with the thiol to form thiyl radicals that initiate polymerization and the nucleophilic addition of thiols to the acrylate double bond. This occurs through the generation of radicals, resulting in a ground-state charge transfer complex.^24^ Esfandiari et al. found an excellent result for ideal storage conditions for thiol-enes employing the buffering capabilities of phosphonic acids and the radical stabilizer 4-methoxyphenol (MeHQ).^38^ The weak acid PPA prevents pH from rising and forces the thiol to switch back from the thiolate anion to thiol (Figure 4).

**Figure 4:**
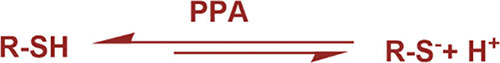
Shifting equilibrium from thiolate anion to thiol using PPA.

From Tables 1a and 1b, it is clear that the pot lives have been increased significantly after the addition of stabilizers to the formulations. For the thiol-epoxy system, just PPA was used to enhance the pot life by suppressing thiol deprotonation (Figure 4). Specifically, it extended the pot life from 20 days to one month for the BADGE-TT1 formulation and from one month to two months for the TMPTE-TT1 formulation. The pot life of the Ebecryl 605-TT1 system was found to be lower than that of other thiol-acrylate systems. We do not have an explanation for this observation, but we do note that Ebecryl 605 has two −OH groups on its primary backbone.

Ammonia catalyzed thiol-epoxy and thiol-acrylate coating curing reaction mechanisms are demonstrated in Figure 5a and 5b. The figures show only dimer structures. Ammonia acts as a base catalyst, deprotonating thiol groups to generate thiolate anions, which are highly nucleophilic. These thiolate anions attack electron-deficient carbons in epoxide rings and acrylates, triggering step-growth polymerization. The reactivity and yield of the thioether product in the base-catalyzed thiol-Michael addition reaction are influenced by factors such as base catalyst strength and concentration, acidity of the thiol, steric accessibility of the thiol, and the nature of the electron-withdrawing group on the C–C bond, epoxy carbon, and functionality of thiol, acrylate, and epoxy resins.^42–46^ In both the commercial and in situ ammonia curing techniques, thiol-acrylates were observed to cure faster than the thiol-epoxy system, as shown in Figure 6a and 6b. Figure 6a shows the cure time of thiol with different acrylates and epoxies that were cured by a 30% aqueous solution of ammonia. All the thiol-acrylate coating compositions take similar amounts of time to cure using 20% (w/w) acidic urea solution (pH ∼4) sprayed onto the surface. The epoxy-acrylate (Ebecryl 605-TT1) formulation cured the fastest. In contrast, the epoxy-thiol (BADGE-TT1) formulation took approximately five times longer to cure than the acrylate-thiol coatings. The TMPTE-TT1 formulation required about three times longer to cure compared to the acrylates when using a 30% aqueous ammonia solution.

**Figure 5:**
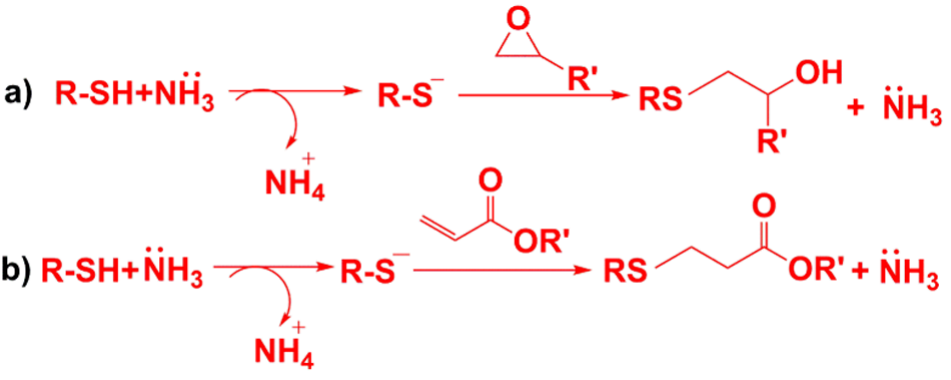
Ammonia catalyzed coating’s curing reaction mechanisms: a) thiol-epoxy and b) thiol-acrylate. R indicates the rest of the TT1 backbone and R′ indicates the rest of the epoxy and acrylates.

**Figure 6:**
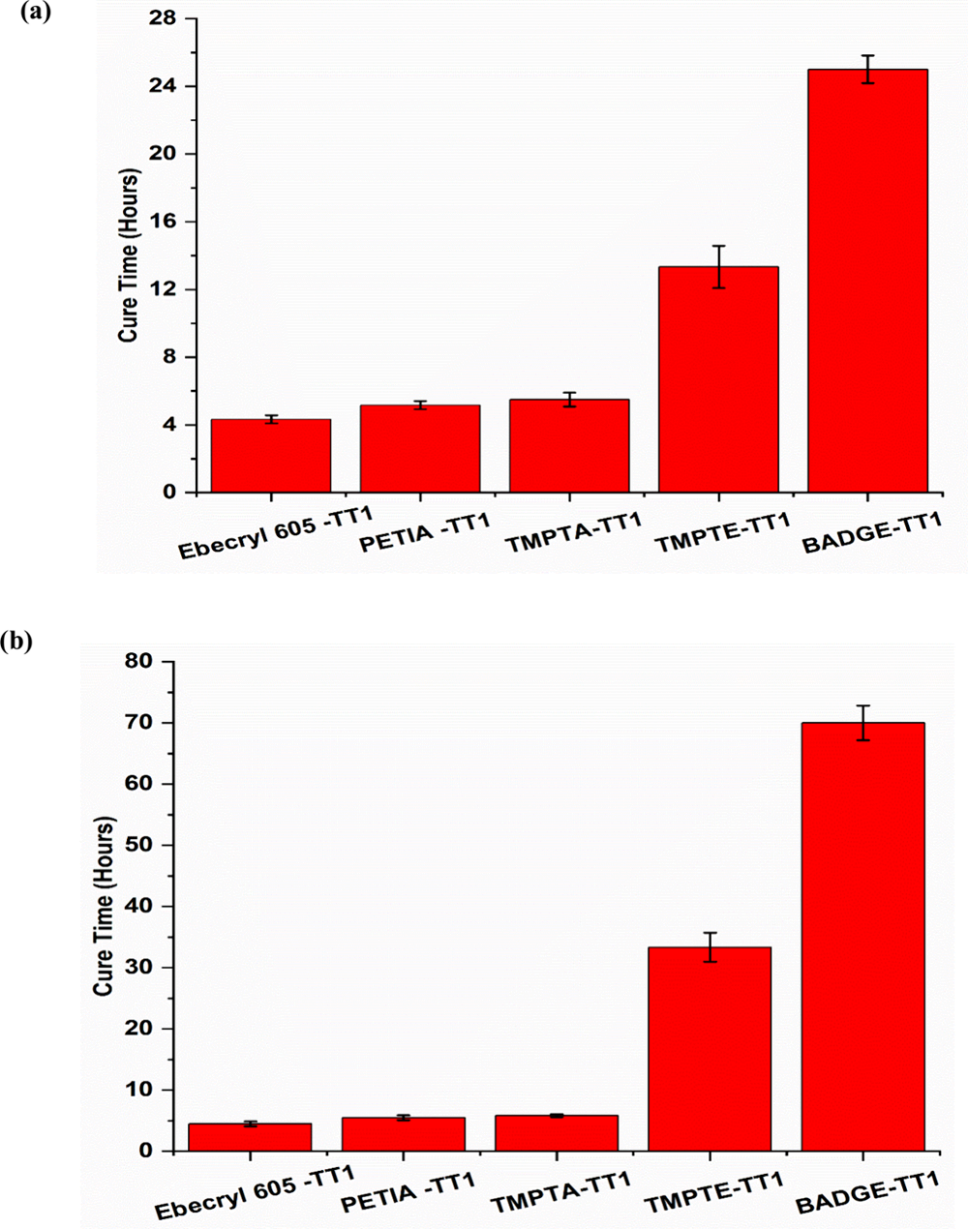
(a) Curing time of thiol-acrylate/epoxy coatings using a 30% NH_3_ solution. (b) Curing time of thiol-acrylate/epoxy coatings using in situ ammonia from the urea-urease (WMSP) reaction.

From Figure 6b, it is evident that the cure time of thiol-acrylate coatings catalyzed by in situ ammonia from the urea-urease reaction is somewhat longer than the time taken by the 30% ammonium hydroxide solution. The cure time for in situ ammonia catalyzed TMPTE-TT1 coatings takes three times longer than 30% ammonia solution catalyst. However, BADGE-TT1 catalyzed by in situ ammonia took 3 days to cure, whereas it took about 1 day using more concentrated 30% aqueous ammonia.

The reason for the different curing times among the acrylates could be explained by the hydroxyl groups at the resin’s backbone structure and bond-breaking energy. Ebecryl 605 resin contains two free hydroxyl groups that can increase hydrogen bonding and thus help decrease the curing time of the coatings. PETIA has one pendant −OH group, but TMPTA has no such −OH group in its structure. While the inclusion of a hydroxyl group appears to improve the reactivity of Ebecryl 605 and PETA in comparison to TMPTA, it is also plausible that the increased polarity of the PETA system generated by the alcohol raises the reaction rate.^47^ Kilambi et al. found that, when electronegative functional groups are added to the thiol-acrylate system, intramolecular effects dominate in the enhancement of acrylate reactivity.^47,48^

Breaking the C—O sigma bond of the diepoxy compound (BADGE) requires more energy than the acrylic C=C pi-bond to form a new S—C polymeric bond with the trithiol (TT1).^49^ Furthermore, when functionality rises, crosslinking increases as well, hastening the curing process.^50^ For example, from Figure 6a and 6b it is evident that curing TMPTE-TT1 is faster than BADGE-TT1.

Some trials were conducted at low and high temperatures to observe the curing status of some coating compositions. At a temperature of −5 °C, all the samples mentioned above exhibited curing in the upper half (approximately 0.5 mm) within 24 h for the acrylates, while the lower section remained uncured. The same thing happened with the epoxy, because diffusion of ammonia gas could be hampered at such a low temperature.^51^ However, coatings at 5 °C required twice as long to cure as at room temperature. Thiol-epoxy samples did not cure quickly; however, thiol-acrylate samples cured quickly at 50 °C, possibly due to radical polymerization of acrylates.

We successfully introduced several ways to produce in situ ammonia using urea, water, and WMSP to catalyze thiol-acrylate and thiol-epoxy reactions (Table 2).

**Table 2:** Comparison of Curing with Ammonia Urea, Water and WMSP

a) Thiol-Acrylate		
Mixing with Resins (Thiol-Acrylate)	Spraying on Top of Applied Coating	Status
Urea and WMSP	DI water or ∼3–4 pH DI water	Worked and cured in usual time
Urea	WMSP and DI water or ∼3–4 pH DI water	Worked and cured in usual time
WMSP	∼3–4 pH urea solution or ∼9 pH urea solution	Worked and cured in usual time

b) Thiol-Epoxy		
Mixing with Resins (Thiol-Epoxy)	Spraying on Top of Applied Coating	Status
WMSP and urea	DI water or ∼3–4 pH DI water	Does not work
Urea	WMSP and DI water or ∼3–4 pH DI water	Worked and cured in usual time
–	WMSP and ∼3–4 pH urea solution or ∼9 pH urea solution	Does not work
WMSP	3–4 pH urea solution or ∼9 pH urea solution	Worked and cured in usual time

From Table 2 it is understandable that we could produce ammonia in several ways to catalyze thiol-acrylate and thiol-epoxy reactions. While mixing urea with formulations, we had to maintain a proper viscosity to apply coatings comfortably on the substrate. According to Table 2b, mixing WMSP with epoxy formulations did not work; however, dispersing on the surface of resins after application to the substrate was effective. Thiol-acrylate formulations were cured with both methods. Cure time and mechanical qualities are identical with those cured from a 20% (W/W) acidic urea solution, as shown in Figure 4 and Table 2. Experiments were conducted to investigate the activity of WMSP in pot life samples of thiol-acrylate formulations containing WMSP, and it was discovered that the activity of WMSP diminished over time. For example, a 20% (w/w) urea solution spray (pH ∼4) was used to evaluate a TMPTA-TT1 coating sample with 0.1 phr WMSP, yielding a cure time of 5.5 h. When the sample composition was tested after 3 days, it revealed inhomogeneous curing with a cure time of 15 h. The sample was still uncured after 48 h when it was tested again a month later. Another TMPTA-TT1 sample was created by increasing the amount of WMSP to 4.3 phr and tested using a 20% (w/w) urea solution spray (pH ∼4), resulting in a 5.5-h cure time the same day. When the same composition was tested after 15 days, the cure time increased to 15 h. Additional tests on the 30th, 90th, and 150th days showed that the coating cured unevenly, with inhomogeneous curing observed 24 and 48 h after each test.

We investigated whether increasing the urea concentration and WMSP would enhance ammonia production and accelerate the reaction, thereby reducing the cure time of the coating formulations. Urea concentrations ranging from 5% to 54% and WMSP concentrations from 0.5 to 8 phr were tested. The expectation was that higher levels of urea and WMSP in the reaction medium would boost the ammonia production, leading to faster curing. However, no significant change in cure time was observed across the different formulations compared to the 20% urea solution and 1 phr WMSP used in our core study. This outcome may be explained by inhibition of urease activity by the ammonium ions produced in the reaction^52^ and/or inhibition by the high concentration of urea.^53^

### Adhesion on Substrates

Tests were conducted on a variety of substrates, including brick, concrete, aluminum sheets, PVC plastics, copper sheets, balsa wood, primed steel panels, and bare steel panels, to determine the adhesion performance of coatings. All of the thiol-acrylate coatings demonstrated excellent adhesion to all of the substrates except bare steel panels. For thiol-acrylate coatings, bare steel panels experience delamination. Samples of thiol-epoxy demonstrated proper adherence to all substrates, including bare steel panels. An example of how well BADGE-TT1 and TMPTA-TT1 adhered to brick and concrete substrates is shown in Figure 7.

**Figure 7:**
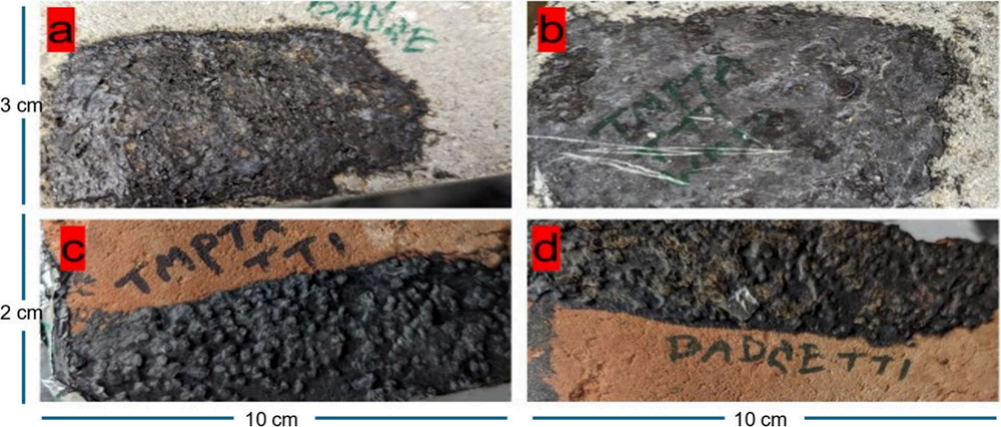
Adhesion of cured coatings on concrete and brick: (a) BADGE-TT1 on concrete, (b) TMPTA-TT1 on concrete, (c) TMPTA-TT1 on brick, and (d) BADGE-TT1 on brick.

### Thiol-Acrylate Conversion

To assess the extent of the major functional group’s conversion, FTIR peak analysis was used to calculate the conversion percentages. Prior to curing thiol-acrylate, the thiol’s S—H stretching frequency and the acrylate group’s C=C stretching frequency for TMPTA-TT1 were 2571 and 1632 cm^–1^, respectively (Figure S1). The fact that the characteristic peaks vanished after curing (shown in Figure S1) indicates full conversion was achieved. For PETIA-TT1, the —SH peak and C=C peak appeared at 2569 and 1634 cm^–1^, respectively. From Figure S2, it is obvious that both peaks disappeared after the curing of this coating formulation. For Ebecryl 605-TT1, the —SH band appeared at 2571 cm^–1^ and C=C is at 1630 cm^–1^. From Figure 8 it is evident that more than 80% conversions of thiol-acrylate functional groups had been achieved after all of the samples of thiol-acrylate coating compositions (TMPTA-TT1, PETIA-TT1, and Ebecryl 605-TT1) hardened. The acrylate C=C and thiol—SH bonds are indicated by yellow and blue color, respectively.

**Figure 8:**
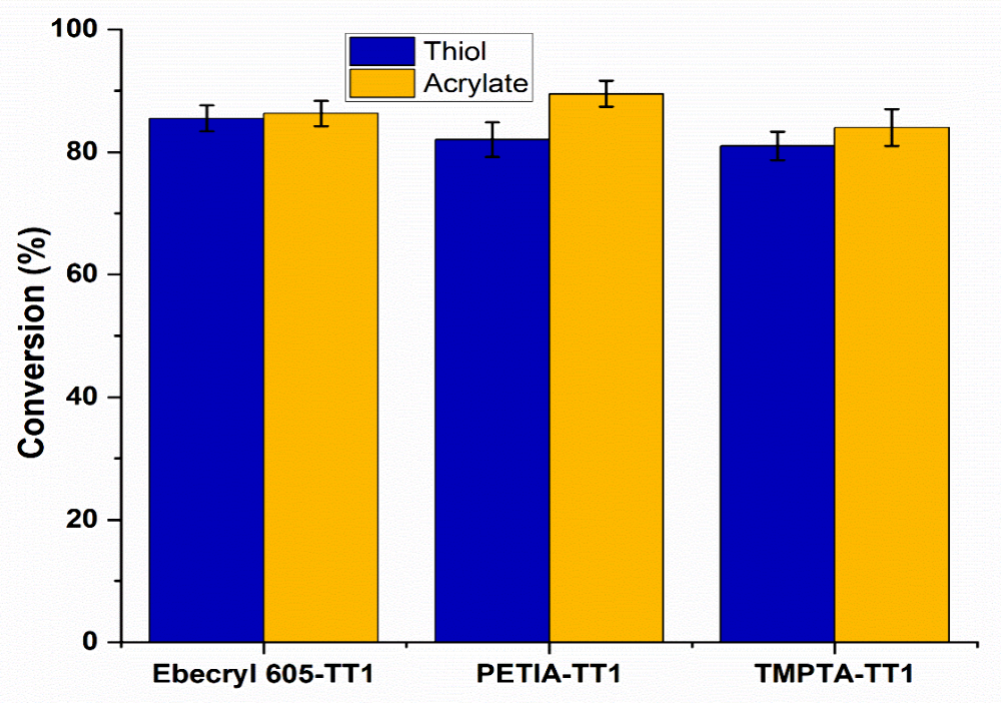
Conversion percentages of major functional group acrylate (C=C) and thiol (—SH) of monomers.

### Thiol-Epoxy Conversion

The thiol’s S–H stretching frequency and epoxy’s C–O stretching frequency for BADGE-TT1 were, respectively, 2569 and 912 cm^–1^ prior to the thiol-epoxy curing (Figure S4). For TMPTE-TT1, thiol’s S–H stretching frequency and epoxy’s C–O stretching frequency for BADGE-TT1 are, respectively, 2569 and 907 cm^–1^ (Figure S5). Once the coatings hardened, all the peaks mostly vanished, suggesting that the monomer had been converted to polymer.

The emergence of a broadband −OH spectrum in the 3100–3600 cm^–1^ region indicates the ring opening reaction of the epoxide group in both BADGE-TT1 and TMPTE-TT1 compositions. The lack of visibility of the newly formed C–S bond IR stretching peak (700–600 cm^–1^) in the thiol-acrylate and thiol-epoxy conversions may be caused by the overlapping of peaks (C–H bending). From the above IR peak analysis (Figures S4 and S5), it is clear that the thiol −SH and epoxy C–O bonds almost completely disappeared after polymerization. The conversion percentages of C–O and −SH for the BADGE-TT1 and TMPTE-TT1 coating compositions exceeded 80% (Figure 9).

**Figure 9:**
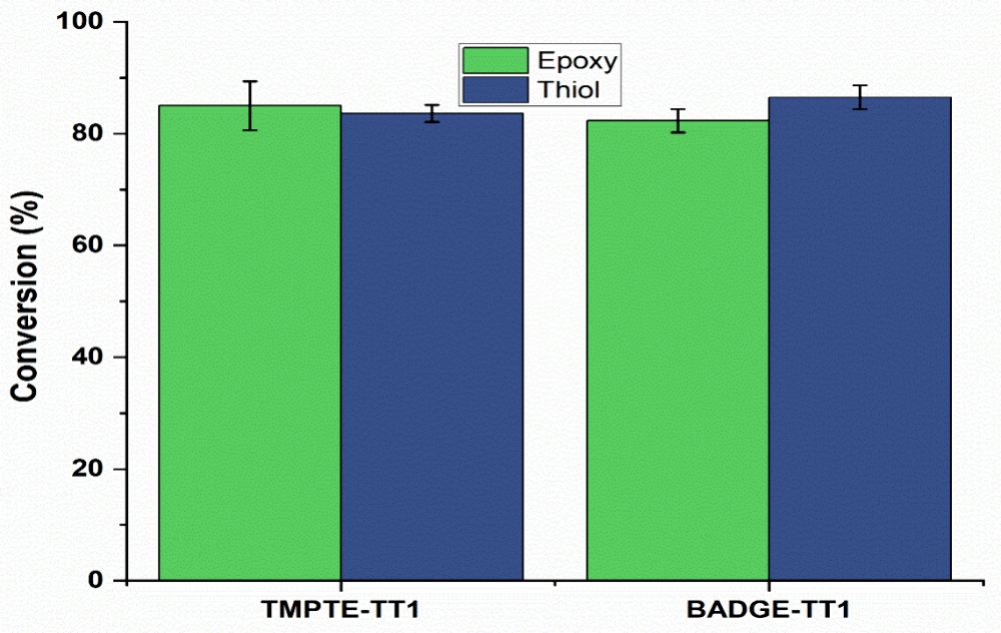
Conversion percentages of epoxy (C–O) and thiol (−SH) of monomers.

## Mechanical Properties

### Pencil Hardness

We conducted pencil hardness analysis according to the ASTM D6336-22 standard and performed scratch and scrape tests using a 2.5 cm chisel by hand. Pencil hardness test results for thiol-acrylate and thiol-epoxy coatings are listed in Table 3.

**Table 3:** Results of Pencil Hardness Testing (ASTM D-6336-22) of Thiol-Acrylate and Thiol-Epoxy Coating Samples

Formulation Resins	Scratch Hardness (Rating)	Gauge Hardness (Rating)
TMPTA-TT1	2H	4H
Ebecryl 605-TT1	2H	4H
PETIA-TT1	2H	4H
TMPTE-TT1	HB	2H
BADGE-TT1	6H	6H

According to the pencil hardness test results, it is obvious that the BADGE-TT1 coating composition exhibited the highest resistance. The scratch and gauge test results reveal that all coating samples, with the exception of BADGE-TT1, performed substantially identically.

The Ebecryl 605-TT1, which contains 25% TPGDA as a diluent, is weaker than the BADGE-TT1. The most likely explanation is that, despite having a backbone with a benzene ring that prevents rotation, the addition of TPGDA increases flexibility and diminishes the polymer’s total strength. As a result, BADGE-TT1 appeared as the strongest coating composition in this pencil hardness study.

### Shore Hardness Test

Five cured coating samples had their hardness tested using a standard handheld digital shore durometer. Figure 10 shows that all the coatings, with the exception of BADGE-TT1, were soft, with durometer scores ranging between 30D and 50D. Because the indenter probe could not pierce through the coating, BADGE-TT1 had the highest durometer rating of 100D. BADGE contains benzene rings in its backbone, which restricts rotation. As a result, BADGE-TT1 exhibited the highest robustness.

**Figure 10:**
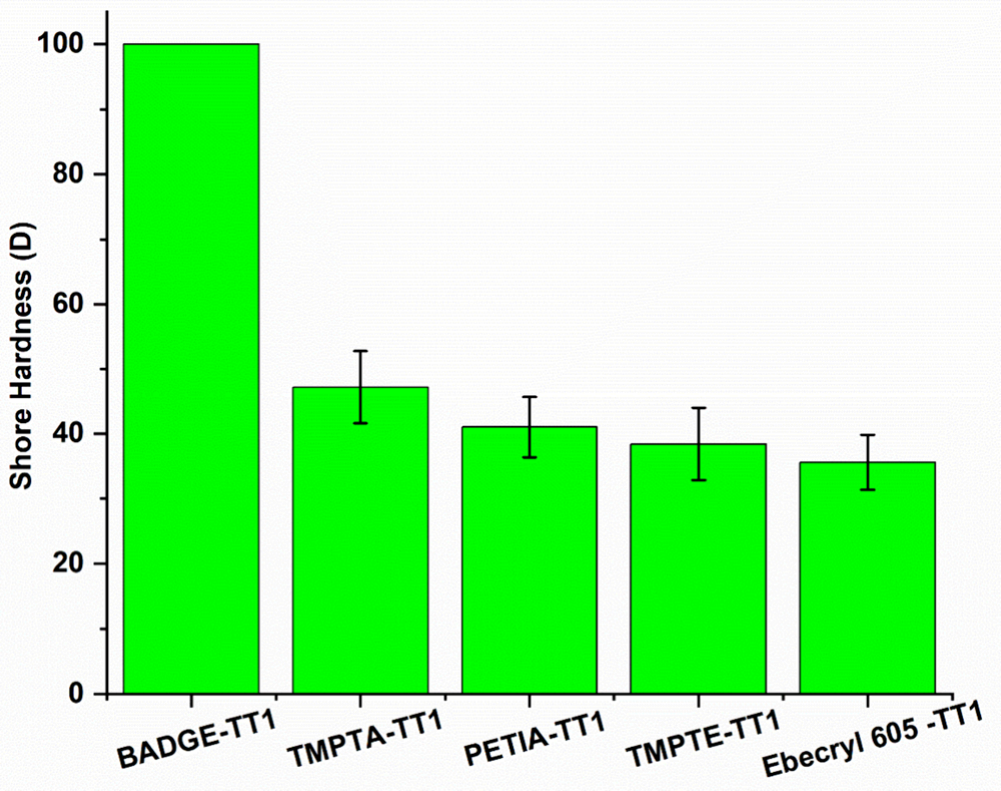
Shore D hardness testing results for three thiol-acrylate and two thiol-epoxy cured coatings.

### Impact Test

We carried out scratch and scrape testing using a 2.5 cm chisel to investigate and compare the mechanical properties for cured coatings. Except for BADGE-TT1, every other system failed the test. A possible explanation could be the chemical structure of BADGE in the BADGE-TT1 coating composition. BADGE has two benzene rings on its primary backbone that resist bond rotation, resulting in a stiff polymeric substance. In the Ebecryl 605-TT1 combination, Ebecryl 605 contains two benzene rings with two hydroxyl groups on its backbone but also includes 25% TPGDA as a diluent, which is likely contributing to the flexibility of the final polymer. If a sample passed the scratch and scrape testing with a chisel, the impact test was performed according to the standard of ASTM G14. The thiol-epoxy BADGE-TT1 coating underwent impact testing, which validated its mechanical strength.

Figure 11a shows a spotless picture of BADGE-TT1 coating before impact test, and Figure 11b demonstrates a spotted coating after impact. After dropping that 1.8 kg weight 25 times on the BADGE-TT1 coating, three nearby impacted links were counted, and the impact score was calculated to be 92.5%, indicating that this coating is mechanically robust.

**Figure 11:**
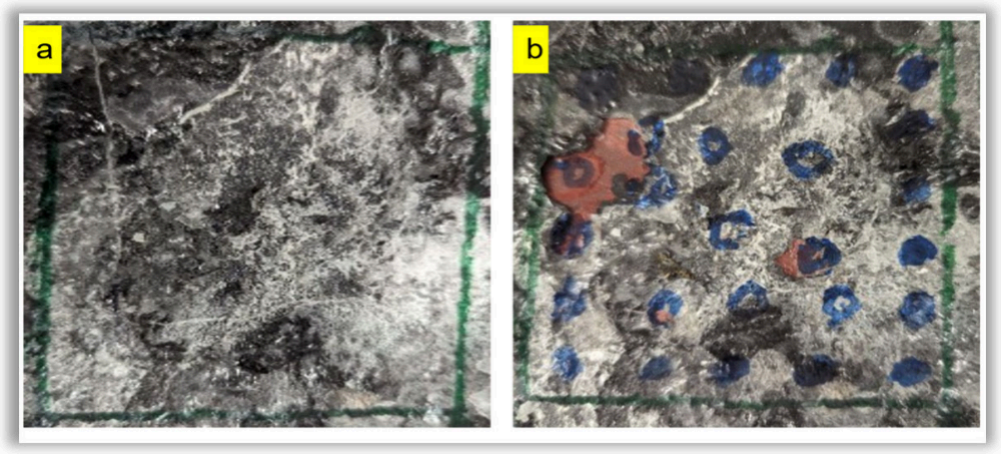
Impact test on a BADGE-TT1 containing coating 3” × 3” substrate marked with blue colored marker. (a) Before impact test. (b) After impact test.

### Cross Hatch Adhesion Test

According to the standard of ASTM D3359, cross hatch tests were performed for every thiol-epoxy and thiol-acrylate coating sample and showed excellent performance. Figure 12 illustrates that BADGE-TT1 exhibited an outstanding performance, achieving the highest rating of 5A.

**Figure 12:**
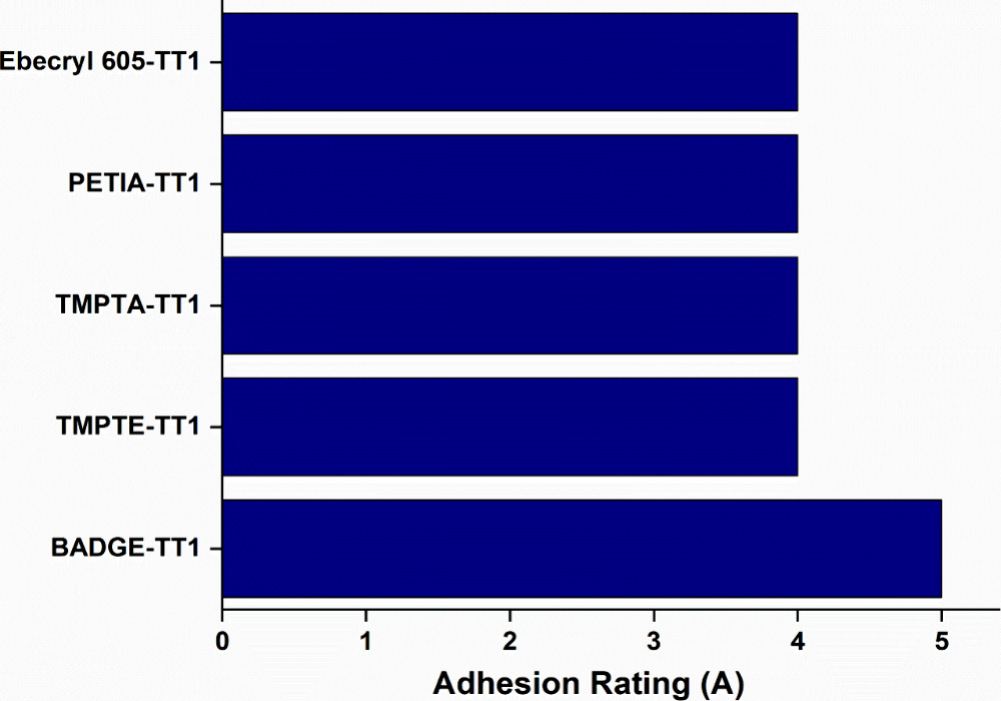
Cross hatch adhesion tape test of thiol-epoxy and thiol-acrylate coatings.

In contrast, the other coating samples displayed minor peeling, receiving a slightly lower rating of 4A.

### Qualitative Chemical Resistance Test

The coatings did not exhibit any softening or delamination when they were immersed in the different fluids. There were no variations in these qualities between the sections that were submerged and those that were not. Table 4 illustrates the outcomes using various chemicals; the coatings are chemical resistant.

**Table 4:** Results of Qualitative Chemical Resistance Testing of All Coating Samples

Chemical	Submerged Time	Result
Ethanol	24 h	Passed
Deicing/defrosting fluid	24 h	Passed
Motor oil	4 weeks	Passed
Natural seawater	4 weeks	Passed

### Thermal Stability Test

Thermal stability was investigated using Thermogravimetric Analysis (TGA). All the coating compositions like TMPTA-TT1, PETIA-TT1, Ebecryl 605-TT1, BADGE-TT1 and TMPTE-TT1 underwent a rapid but small mass loss (∼2%) at around 100 °C. The TGA graphs for all the cured coating samples are shown in Figure S6. Almost all of the sample compositions experienced a fairly large weight loss (30–40%) at around 330 °C (Table S1). This significant degradation may be attributed to S–C bond breakdown; it is clear that these coating compositions demonstrated significant heat stability in a nitrogen atmosphere. Additionally, all cured coating samples were placed in an oven at 50 °C for two months, and the coatings on the substrate showed no change from the previous condition after performing scratch testing.

## Conclusion

Aqueous ammonia and ammonia produced in situ both worked as catalysts for cure-on-demand thiol-epoxy/thiol-acrylate coating curing reactions. Short pot lives of the coating formulations were overcome by adding stabilizers. Thiol-epoxy coatings prepared with BADGE were mechanically stronger than thiol-acrylate systems, but the cure time of acrylates was much shorter than that of epoxies. The pot life stabilization and the subsequent curing of resins were done successfully by using ammonia gas for the first time. The utilization of watermelon seed powder as a source of urease enzyme to produce ammonia into the system can be environment and user friendly by reducing the loss of ammonia to the environment. Another notable advantage is that the reaction time can be controlled by utilizing the autocatalytic mechanism of the urea-urease reaction. The disadvantage is the softness of the thiol-acrylate coatings, which could be improved in future investigations.

## Supplementary Material



## Notes

FTIR spectra of thiol-acrylate coatings (Figures S1–S3); FTIR spectra for thiol-epoxy coatings (Figures S4 and S5) (these spectra confirm the characteristic functional group transformations associated with the curing reactions; and thermogravimetric analysis (TGA) results, illustrating the thermal decomposition profiles of both thiol-acrylate and thiol-epoxy coating compositions (Figure S6 and Table S1) (PDF)
