# Supplementary material for: A Novel Approach to Cure-on-Demand Coatings Using Ammonia to Catalyze Thiol-Acrylate and Thiol-Epoxy Reactions
Source: ACS Omega. 2025 May 15;10(20):20417–26. doi: 10.1021/acsomega.5c00523 (PMC12120628; doi:10.1021/acsomega.5c00523)
Supplement: Supplementary file 1 [file ao5c00523_si_001.pdf]

Supporting Information (SI)

A novel approach to cure-on-demand coatings using ammonia to catalyze thiol-acrylate and thiol-epoxy reactions

Md Abdullah Al Mahmud, John A. Pojman*

Department of Chemistry and the Macromolecular Studies Group, Louisiana State University, Baton Rouge, Louisiana, USA

*John A. Pojman

Department of Chemistry and the Macromolecular Studies Group, Louisiana State University, Baton Rouge, LA 70803, USA.

Email: john@pojman.com

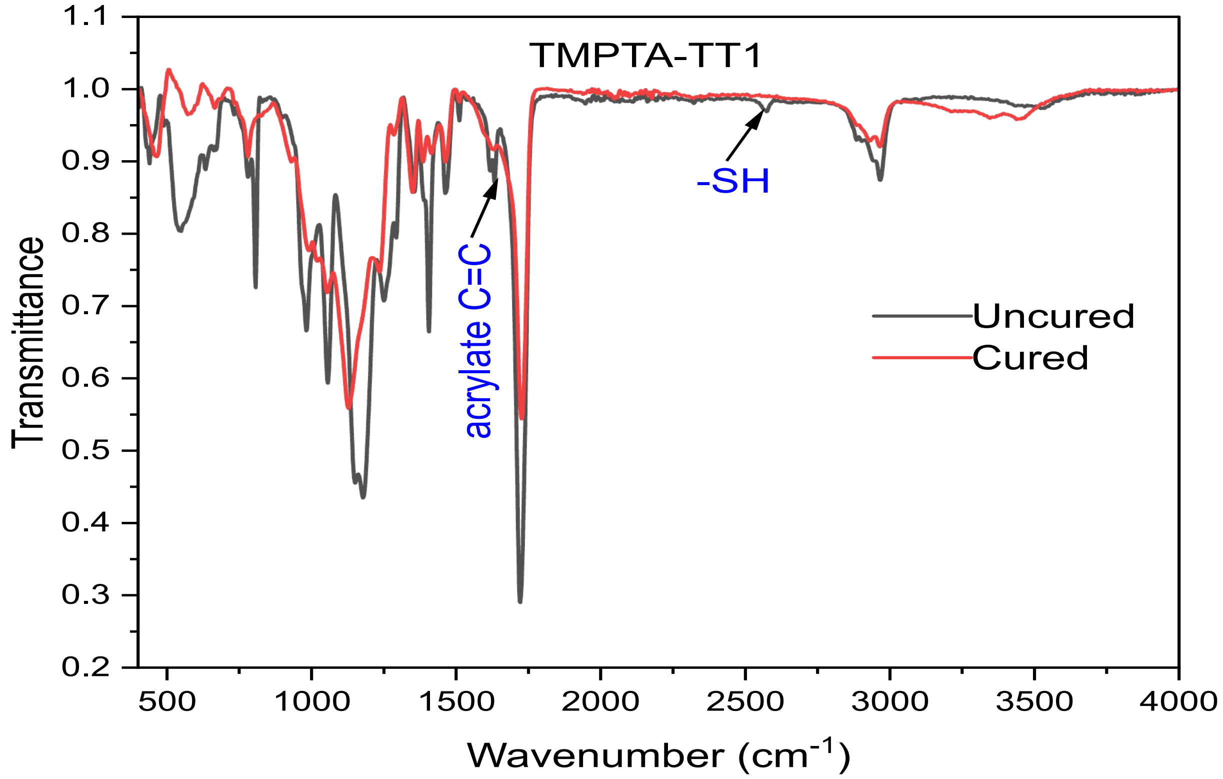

Figure S1. IR stretching bands of major thiol-acrylate functional groups before and after curing TMPTA-TT1 coating composition.

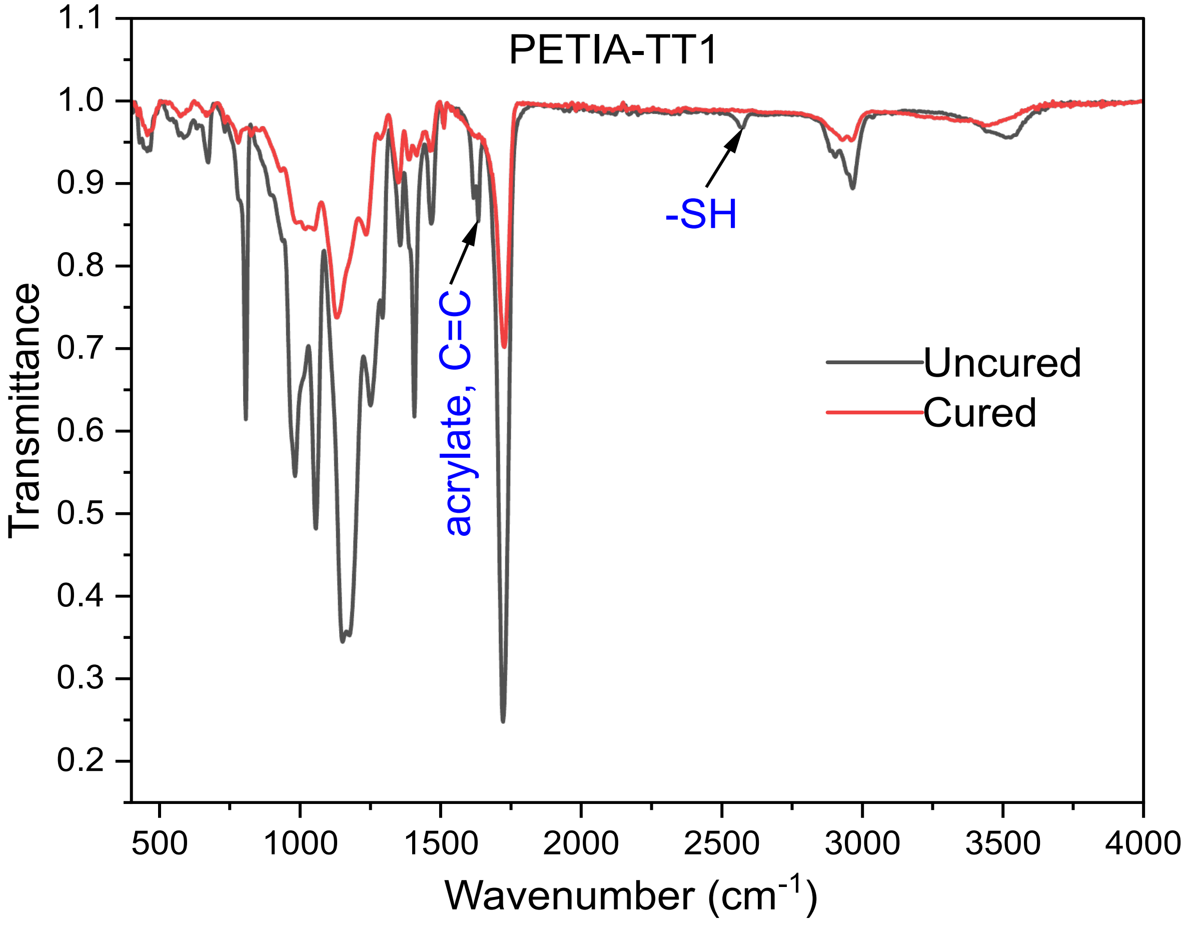

Figure S2. IR stretching bands of major thiol-acrylate functional groups before and after curing PETIA-TT1 coating composition.

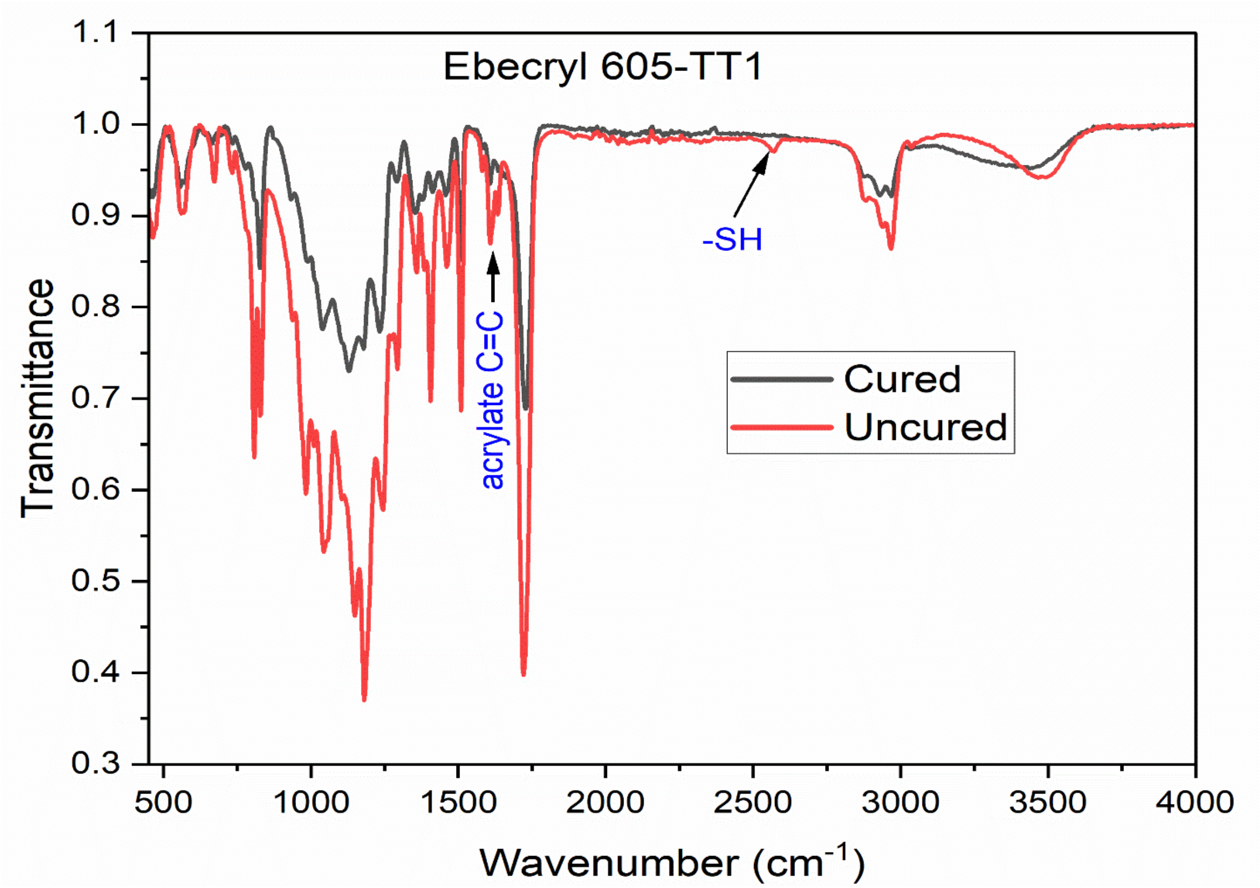

Figure S3. IR stretching bands of major thiol-acrylate functional groups before and after curing Ebecryl 605 -TT1 coating composition.

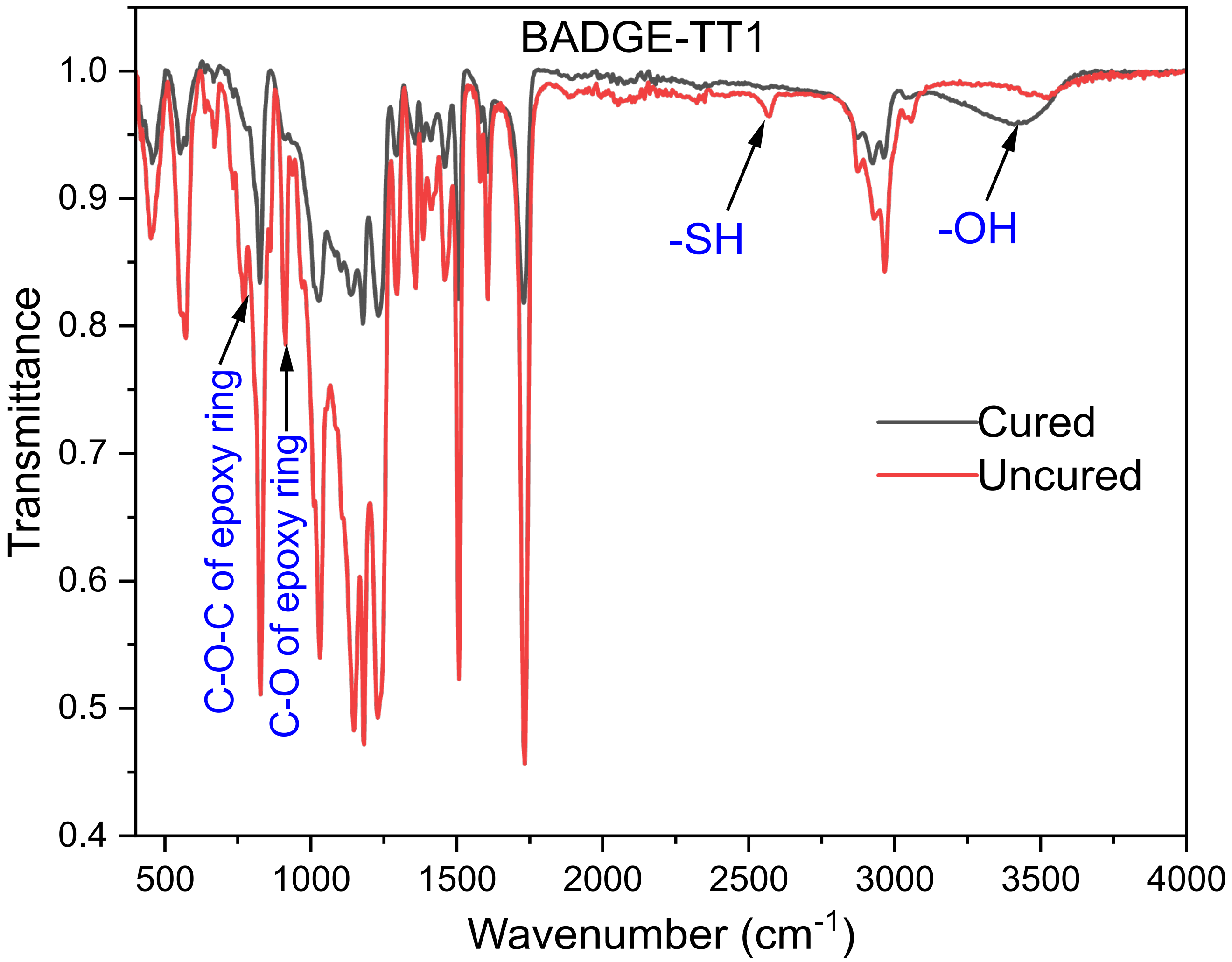

Figure S4. IR stretching bands of major thiol-epoxy functional groups before and after curing BADGE-TT1 coating composition.

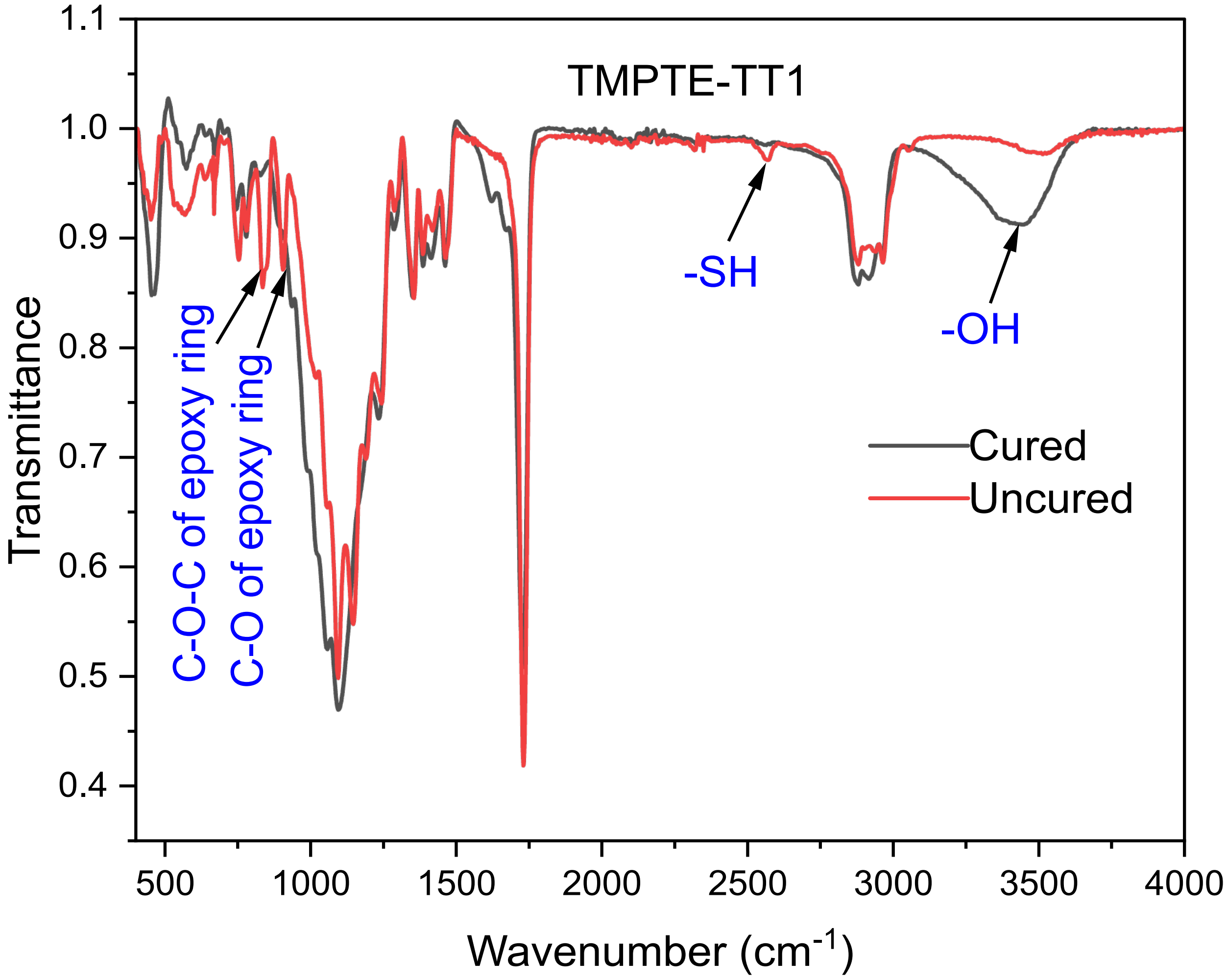

Figure S5. IR stretching bands of major thiol-epoxy functional groups before and after curing TMPTE-TT1 coating composition.

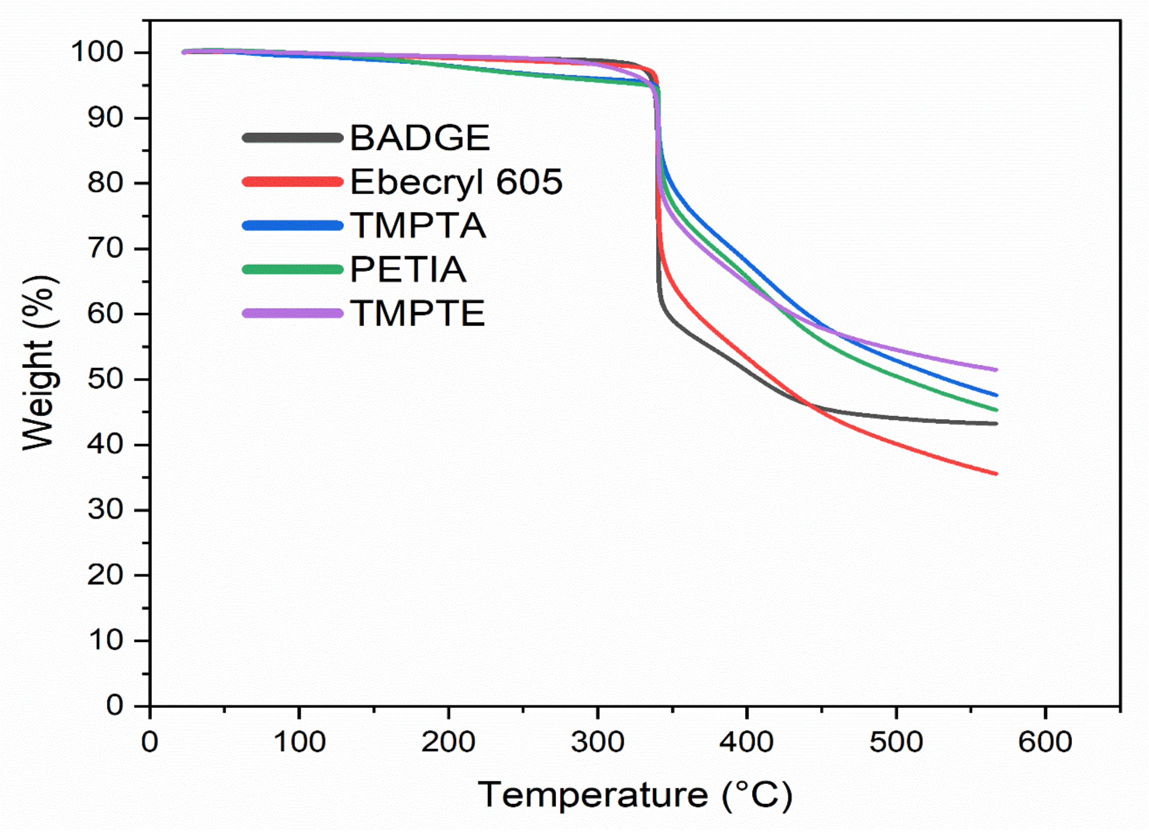

Figure S6. TGA analysis for thermal decomposition of cured thiol-acrylate and thiol-epoxy coatings at a heating rate of 50°C min^{-1} under N_2 flow of 100 ml min^{-1}.

Table S1. T-onset determined from TGA graph for all thiol-epoxy and thiol-acrylate coatings.

**Cured Coatings**	**T-onset**
BADGE-TT1	340°C
TMPTE-TT1	298°C
TMPTA-TT1	338°C
PETIA-TT1	350°C
Ebecryl 605-TT1	337°C
